# Leukocytosis at Presentation Is an Independent Predictor for Hemorrhage in Cerebral Cavernoma

**DOI:** 10.3390/diagnostics16081214

**Published:** 2026-04-18

**Authors:** Harun Asoglu, Tim Lampmann, Johannes Wach, Mohammed Banat, Marcus Thudium, Hartmut Vatter, Erdem Güresir, Motaz Hamed

**Affiliations:** 1 Department of Neurosurgery, University Hospital Bonn, Venusberg-Campus 1, 53127 Bonn, Germany; tim.lampmann@ukbonn.de (T.L.); johannes.wach@uniklinik-leipzig.de (J.W.); mohammed.banat@ukbonn.de (M.B.); hartmut.vatter@ukbonn.de (H.V.); erdem.gueresir@uniklinik-leipzig.de (E.G.); motaz.hamed@ukbonn.de (M.H.); 2 Department of Anaesthesiology and Intensive Care Medicine, University Hospital Bonn, Venusberg-Campus 1, 53127 Bonn, Germany; marcus.thudium@ukbonn.de

**Keywords:** CCM, diagnostic, hemorrhage, epilepsy, neuroinflammation

## Abstract

**Objective**: Cerebral cavernous malformations (CCMs) are usually occult but can present with a symptomatic hemorrhage. Treatment recommendations for CCMs are still controversially discussed, as all CCMs have signs of chronic hemorrhage. The distinction of acute hemorrhage can be difficult, especially when patients only present with mild symptoms. Because of emerging evidence supporting inflammatory burden as a main avenue in the disease pathogenesis of CCMs, the aim of the present study was to investigate routine inflammatory parameters to support decision-making in ambiguous cases. **Methods**: A total of 87 patients who underwent CCM resection at the authors’ institution between 2008 and 2021 were included in this study. Data were recorded retrospectively. Patients were dichotomized into two groups: those with acute hemorrhage and those without, as a control group (e.g., resection for seizure control). Inflammatory parameters included C-reactive Protein (CrP), White Blood Cell Count (WBC), Red Cell Distribution Width (RDW), and Mean Platelet Volume/Platelet Count Ratio (MPV/PC). **Results**: The receiver operating characteristic curve demonstrated moderate diagnostic accuracy for predicting acute hemorrhage from CCM based on WBC at admission (AUC: 0.74, 95%-CI: 0.63–0.84) with a cut-off of ≥6.595 G/L. The multivariable analysis confirmed that having a WBC > 6.595 G/L is an independent predictor for acute hemorrhage of CCM (adjusted odds ratio: 4.5, 95%-CI: 1.8–11.2, *p* < 0.001). **Conclusions**: A white blood cell count >6.595 G/L was significantly associated with acute hemorrhage in CCMs and appears to be a quick-to-use biomarker in controversial cases. Moreover, leukocytosis emphasizes the involvement of neuroinflammation in acute hemorrhage of CCM. Further investigations are needed to analyze the precise role of inflammation in CCM pathogenesis and its impact on treatment strategies.

## 1. Introduction

Cerebral cavernous malformations (CCMs) are vascular malformations consisting of hyalinized, enlarged capillaries with no intervening brain parenchyma, and they are surrounded by hemosiderin deposits and reactive gliosis [1,2,3,4,5]. CCMs can occur sporadically or in familial forms associated with autosomal-dominant mutations in *CCM1*, *CCM2*, or *CCM3* genes, which play crucial roles in endothelial junction stability and angiogenic signaling [6]. The prevalence of CCMs in the general population is about 0.5%, and they account for 10–25% of all cerebral vascular malformations [1,2,6,7,8,9,10]. CCMs are usually occult but can present with a symptomatic hemorrhage, mostly between the second and fifth decades of life [11,12]. The annual bleeding rate ranges from 0.25 to 6%, but bleeding risk depends on many factors [1,12,13,14,15,16]. For example, in familial CCMs, the bleeding risk is twice as high as in sporadic CCMs [17]. In cases of previous bleeding, the risk of hemorrhage increases up to 4.5–22.9% per year. Depending on the localization of the CCM, the bleeding risk varies; e.g., brainstem CCMs have a higher bleeding risk than supratentorial CCMs [11]. Furthermore, due to the location (supratentorial vs. infratentorial), hemorrhage results in different neurological disabilities and outcomes [18]. The primary symptoms of CCM hemorrhage include headache, focal neurological deficits, and epilepsy [12]. Histopathologically, CCMs lack components of vascular structures, like smooth muscle cells and basal membrane integrity. This fragility leads to chronic leakage and microhemorrhages, which can trigger local immune responses [6]. The repeated breakdown of blood components leads to perilesional hemosiderin deposition, which is linked to seizure activity and progressive tissue dysfunction [6]. This blood degradation cycle creates a chronic local inflammation, which could lead to lesion instability and to progression with secondary neurological deficits.

Treatment recommendations for CCMs are still controversially discussed. Main reasons for a treatment are (1) recurrent hemorrhage with neurological deficits, (2) insufficient seizure control with drugs and CCM-related epilepsy, and (3) growth [19]. All CCMs have signs of chronic hemorrhage. The distinction of acute hemorrhage can be difficult, especially when patients only present with mild symptoms.

The connection between inflammatory responses and hemorrhagic events has been described in various cerebrovascular anomalies, such as aneurysms, cerebral arteriovenous malformations, and cerebral amyloid angiopathy [20,21,22,23,24]. Yau et al. showed in a CCM mouse model an increased expression of inflammation-related genes in endothelial cells [25]. Furthermore, an infiltration of neutrophils into the CCM was also found [25]. Lai et al. showed that CCM endothelial cells exhibit elevated expression of inflammatory pathways [6]. These include *NLRP3* inflammasome components and COX-2, which promote the release of pro-inflammatory cytokines and chemokines [5,6]. This expression profile allows the recruitment of immune cells, such as microglia, macrophages, neutrophils, and T lymphocytes, into and around CCM lesions and, therefore, creates an inflammatory milieu [6]. Additionally, recent work has emphasized a significant role for reactive astrocytes in CCM regions. Astrocytes respond to hemorrhagic and vascular stress by upregulating pro-inflammatory mediators and contribute to the disruption of the blood–brain barrier [5]. This astrocytic response amplifies local inflammation and can influence neuronal excitability and thereby link inflammatory processes to clinical symptoms such as seizures [5]. Furthermore, inflammatory activation within lesions is associated with increased hemorrhagic events [5]. Inflammatory cells and mediators contribute to endothelial damage and the destabilization of the microvascular system. This increases the risk of clinically significant bleeding and the occurrence of neurological deficits [5].

Because of emerging evidence supporting inflammatory burden as a main avenue in the disease pathogenesis of CCMs [26], the aim of the present study was to investigate routine inflammatory parameters to support decision-making in ambiguous cases.

## 2. Materials and Methods

Consecutive patients who underwent CCM resection at the authors’ institution between 2008 and 2021 were included in this study. Additional inclusion criteria were an age of over 18 years, histopathological confirmation of CCM, availability of follow-up data, and a complete medical history. Patients who did not meet these criteria were excluded from the analysis. The data were collected retrospectively from patients undergoing CCM resection who presented with neurological deficits. For these patients, rehabilitation was recommended between the onset of symptoms and surgical resection in order to reduce perioperative morbidity. The duration of rehabilitation was at least four weeks. Patients were dichotomized into two groups: those presenting with acute hemorrhage and those without, serving as a control group (e.g., patients undergoing resection primarily for seizure control). Patients in the control group were carefully evaluated by the Department of Epileptology, and only individuals whose seizures were confirmed to originate from the CCM were offered surgical removal and included in the study. Laboratory parameters were assessed at the time of acute bleeding for patients in the hemorrhage group (taking into account the rehabilitation period between symptom onset and resection) or for those in the control group who had undergone prior surgery. Peripheral blood samples for the acute group were obtained within 24 h of clinical presentation and prior to any surgical or rehabilitative procedures. Inflammatory parameters evaluated included C-reactive Protein (CrP), White Blood Cell Count (WBC), Red Cell Distribution Width (RDW), and Mean Platelet Volume/Platelet Count Ratio (MPV/PC). A total of 87 patients met all inclusion criteria and were included in the final analysis.

The statistical analyses were performed using SPSS Statistics (Version 29, IBM Corp., Armonk, NY, USA). For parametric data, the independent *t*-test was applied to compare group means, while Fisher’s exact test was used for categorical variables. The optimal cut-off values for parametric variables were determined using Youden’s index in receiver-operating characteristic (ROC) analysis. Multivariable analysis was conducted to identify independent risk factors associated with acute hemorrhage in CCMs. A *p*-value of less than 0.05 was considered statistically significant.

## 3. Results

Patients who underwent CCM resection had a mean age of 40 years, with an almost equal distribution between female and male patients. 44 (50.6%) patients suffered from acute hemorrhage. Baseline characteristics and routine inflammatory parameters of both groups are shown in Table 1 and Table 2, respectively. While the baseline characteristics were comparable between the groups, a higher WBC was significantly associated with acute hemorrhage in CCM patients. Other inflammatory parameters like CrP, RDW, and MPV/PC did not show a significant connection to an acute hemorrhage. 

**Figure 1:**
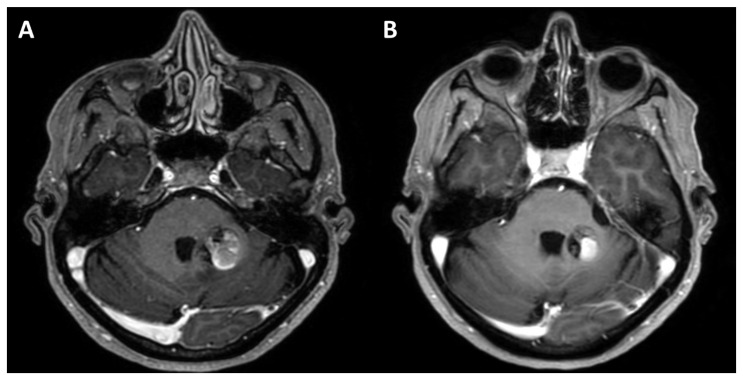
Axial MR images of acute CCM hemorrhage (**A**) after chronification (**B**) before surgery.

Additionally, Figure 1 provides visual insight through exemplary magnetic resonance imaging (MRI) scans, illustrating the same patient’s CCM during an acute hemorrhage and subsequently, six weeks later, prior to surgery.

**Figure 2:**
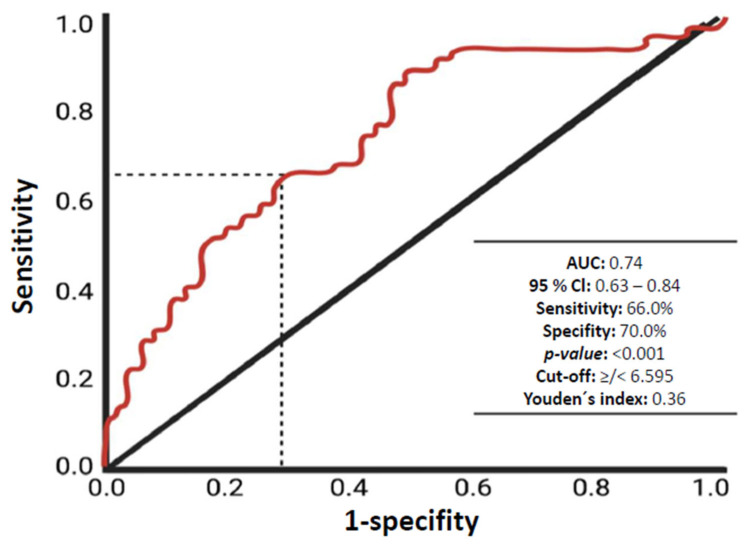
The receiver operating characteristic (ROC) curve shows the white blood cell count in the prediction of cavernoma’s bleeding risk.

**Figure 3:**
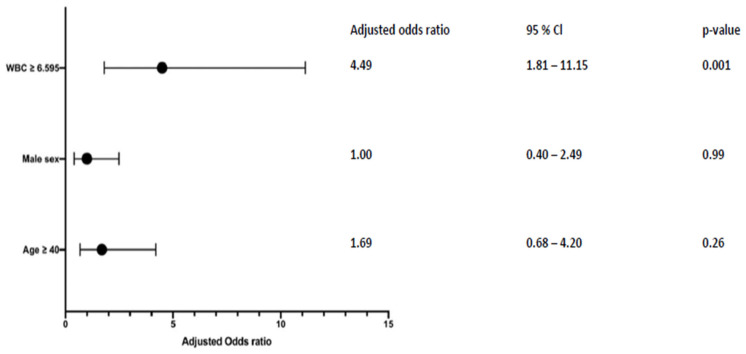
Multivariable analysis of risk factors for acute CCM hemorrhage with regard to white blood cell count (WBC), sex, and age.

The ROC curve demonstrated moderate diagnostic accuracy for predicting acute hemorrhage from CCM based on WBC at admission (AUC: 0.74, 95%-CI: 0.63–0.84) with a cut-off value of ≥6.595 G/L as shown in Figure 2. Based on these findings, we conducted a multivariable analysis to investigate whether WBC is an independent factor while accounting for demographic variables such as sex and age. The results presented in Figure 3 confirmed that having a WBC > 6.595 G/L is an independent predictor of acute hemorrhage in CCMs (adjusted odds ratio: 4.5, 95%-CI: 1.8–11.2, *p* < 0.001).

## 4. Discussion

In many cases, the treatment of acute hemorrhage in CCMs with neurological deficits and an appropriate radiological location of the CCM is clear. Those patients are offered surgical removal or a conservative treatment with a follow-up, depending on the severity of the disease, location, and bleeding. However, in some cases, the neurological conditions and radiological findings are divergent. In these cases, we aimed to identify an independent objective parameter to support decision-making if the acutely occurring deficits correlate with an acute hemorrhage of CCM.

The radiological classification of CCMs, as proposed by Zabramski, categorizes these lesions into four distinct types [27,28]. Type 1 CCM is characterized by acute or subacute hemorrhage. Pathologically, these lesions demonstrate subacute hemorrhage surrounded by hemosiderin-laden macrophages, as well as gliotic brain tissue [27,28]. This pattern reflects both the acute injury and the ongoing healing process, with hemosiderin deposition serving as a marker of prior hemorrhage. Type 2 CCMs represent the classic “popcorn”-like lesion. Often, a hemosiderin ring is observed, which correlates with intracavernous hemorrhage and thrombosis. The surrounding brain tissue is gliotic and stained with hemosiderin [27,28]. These lesions tend to reflect a more chronic bleeding pattern and are often easier to identify due to their characteristic imaging appearance. Type 3 CCMs are typically associated with chronic hemorrhage and hemosiderin deposition [27,28]. This type indicates that the lesion has undergone multiple bleeding episodes, resulting in substantial gliotic changes in the surrounding brain tissue. Lastly, type 4 CCMs differ in that they are only visible on hemosiderin-sensitive sequences [28]. These lesions are often small and asymptomatic, making them easily overlooked on conventional MRI sequences. Overall, all four types of CCMs are associated with hemosiderin deposition. This can make it challenging, in some cases, to distinguish between acute, subacute, and chronic hemorrhage as well as to correlate imaging findings with the patients’ clinical symptoms. Saari et al. reported an association between patients’ clinical symptoms and type 1 CCMs [28]. They also described the potential regression of type 1 CCMs into asymptomatic type 2 or type 3 lesions over time [28]. Wang et al. evaluated the predictive value of the Zabramski classification and found that type 1 CCMs were associated with a higher risk of rebleeding, which in turn correlated with subsequent clinical symptoms [27]. These findings suggest that in many cases, the MRI results are sufficient and correlate well with the patient’s clinical symptoms. However, there are situations in which MRI findings alone are not informative enough to establish a clear link between lesion type and clinical course. In such cases, additional markers or diagnostic parameters are necessary to more accurately assess disease activity and better predict potential risk for the patient.

Our results show that an increased white blood cell count (>6.595 G/L) correlates with an acute hemorrhage of a CCM. It is well-established that injuries trigger leukocytosis as part of the inflammatory response [29]. In line with this, an acute hemorrhage of a CCM represents a form of brain injury and is therefore likely to induce a neuroinflammatory response [30]. This response may involve activation of microglia, recruitment of peripheral immune cells, and the release of proinflammatory cytokines, all of which can contribute to an elevation in circulating leukocyte levels.

Leukocytosis can result from various factors, including infection, trauma, medication, smoking habits, etc., [29,31]. To account for potential confounders, we analyzed preexisting diseases within our study cohort. None of our study patients had any known connective tissue disorders or blood-borne diseases, allowing us to largely exclude leukocytosis due to underlying chronic conditions. Medication such as dexamethasone, which is frequently used to treat cerebral edema and is known to cause an aseptic leukocytosis, was only taken by one patient in our study cohort. The majority of patients did not receive glucocorticoids of any kind. Therefore, it is unlikely that medication alone significantly influenced our findings or limited the validity of the observed association. Another potential contributor to leukocytosis is smoking [31], as it has been shown that smokers tend to exhibit elevated baseline white blood cell counts. In our study, four patients were identified as smokers; however, no significant difference in leukocyte levels was observed between smokers and non-smokers. This suggests that smoking did not substantially confound our results.

Infections can also cause leukocytosis. Therefore, we analyzed the amount of CrP on the day of their arrival. In both groups, CrP levels did not show significant changes. It is commonly known that CrP is an acute-phase protein that increases in an inflammatory response [32]. According to our hypothesis that acute hemorrhage of CCM causes an inflammatory response in the surrounding area, an increase in CrP would further support this assumption. However, CrP is also known for a delayed elevation following an inflammatory stimulus [32]. Thus, it is possible that the hemorrhage of CCM triggered a localized inflammatory response, but systemic CrP levels may not yet have risen at the time of measurement. In contrast, the increase in WBC is known to occur more rapidly and may therefore represent an earlier indicator of inflammatory activity [31,32]. The lack of significant CrP elevation likely reflects its slower rise in the acute phase, whereas WBC may increase more rapidly in response to physiological stress and acute intracranial injury. Leukocytosis should be interpreted as a reactive biomarker of the acute hemorrhage rather than a causative factor. The observed elevation likely reflects stress-induced leukocyte demargination following intracranial injury. Another potentially informative parameter would be the blood sedimentation rate, which reflects ongoing inflammation over a longer period; this value was not routinely obtained in our laboratory setting. This represents a limitation of the present analysis, as blood sedimentation rate could have provided complementary information to CrP and WBC levels. An increased WBC and elevated CrP are also commonly observed in every hospital due to infectious processes. In our study, the leukocytosis and CrP values were only slightly elevated and remained within a physiological range. In the presence of a clinically relevant infection, one would typically expect more pronounced pathological elevations. Nevertheless, early stages of infection may initially present with mild leukocytosis within the upper physiological range, while other parameters, such as CrP, require more time to increase. Overall, we analyzed common inflammatory parameters without identifying clear evidence of systemic inflammation. However, it remains unclear to what extent localized neuroinflammation, as might occur in the context of CCM hemorrhage, is reflected by systemic blood markers such as leukocytosis or CrP. It is conceivable that such localized processes remain largely undetected in peripheral blood analyses. Our findings are consistent with the concept of local oxy-inflammation, as proposed by Bianconi et al. (2022), suggesting that chronic sterile inflammation and oxidative stress, particularly in the presence of developmental venous anomalies, may contribute to lesion instability and hemorrhagic susceptibility [33]. In our study, no molecular or histopathological analyses of the resected CCM tissue were performed. Future studies should therefore aim to investigate local inflammatory markers, cytokine expression, and cellular responses within the lesion itself. Such approaches may provide a more detailed understanding of the underlying pathophysiological mechanisms and help to clarify the relationship between systemic and localized inflammatory responses in CCM-associated hemorrhage.

Taken together, these considerations support the assumption that the observed leukocytosis is most likely associated with the acute hemorrhagic event itself rather than external or preexisting factors. Nonetheless, it cannot be completely ruled out that subclinical influences may have contributed to the variability in leukocyte counts. Future studies with larger sample sizes and more detailed patient stratification may help to further clarify the relative contribution of these factors.

In comparison to other vascular diseases of the central nervous system, such as arteriovenous malformations (AVMs) and subarachnoid hemorrhage (SAHs), neuroinflammation is also a highly discussed topic. Despite differences in etiology, acute hemorrhagic event versus congenital vascular malformation, both AVMs and SAH share key aspects of neuroinflammatory pathologies [34,35]. In both conditions, disrupted vascular integrity and exposure to blood components in the brain tissue trigger activation of glial cells, recruitment of peripheral immune cells, and upregulation of pro-inflammatory cytokines [34,35]. These immune responses can exacerbate vascular integrity, neuronal injury, and secondary complications [34,35]. Targeting specific inflammatory pathways, modulating immune cell phenotypes, and stabilizing endothelial function are active areas of translational research that may yield new strategies to reduce hemorrhage risk in CCM patients or other vascular diseases like SAH or AVMs. In SAH patients, a prospective trial was executed to determine whether dexamethasone improves the outcome by its anti-inflammatory effect as a glucocorticoid [36]. The results of the study are highly anticipated.

### Limitations

We acknowledge that our study has some limitations. First, it was a retrospective study with a limited number of patients, which introduces inherent limitations in terms of statistical power and generalizability. Our study exclusively included surgically treated patients, which results in a cohort with severe symptomatic and clinically relevant cases. While this limits generalizability, the findings remain relevant for surgical decision-making. Second, leukocytosis can be influenced by a variety of other medical conditions. Although we made efforts to investigate and exclude most potential confounding factors, it is possible that some conditions were not fully represented in our cohort. Finally, leukocytosis alone is not a sufficient parameter to reliably indicate acute hemorrhage in CCMs. Its interpretation must be considered in the context of clinical presentation and radiological findings. Nevertheless, it may serve as a useful adjunctive marker in cases where the diagnosis remains ambiguous.

## 5. Conclusions

A white blood cell count >6.595 G/L was significantly associated with acute hemorrhage of cerebral cavernous malformations and appears to be a quick-to-assess biomarker in cases where the diagnosis is uncertain. Moreover, the presence of leukocytosis underscores the potential role of neuroinflammation in the pathophysiology of acute CCM hemorrhage. These findings highlight the need for further investigations to elucidate the contribution of inflammatory processes in the pathogenesis of CCMs.

## Figures and Tables

**Table 1:** Baseline characteristics of patients with acute CCM hemorrhage.

	Acute CCM Hemorrhage	No Acute CCM Hemorrhage	*p*-Value
Overall	44 (100%)	43 (100%)	
Mean Age (±SD) [years]	42.1 ± 18.2	37.4 ± 14.6	0.179
Sex			1.000
Female	21 (47.7%)	21 (48.8%)	
Male	23 (52.3%)	22 (51.2%)	
Seizure	11 (25%)	42 (97.7%)	<0.001
Blood-Borne Disease	0 (0%)	0 (0%)	-
Connective Tissue Disorders	0 (0%)	0 (0%)	-
Dexamethason Intake	1 (2.3%)	0 (0%)	1.000
Known Nicotine Abuse	1 (2.3%)	3 (7%)	0.531

SD: Standard Deviation.

**Table 2:** Comparison of inflammatory parameters in association with acute CCM hemorrhage.

	Acute CCM Hemorrhage	No Acute CCM Hemorrhage	*p*-Value
C-reactive Protein (CrP) (±SD) [mg/L]	2.5 ± 5.1	2.2 ± 4.5	0.793
White Blood Cell Count (WBC) (±SD) [G/L]	8.2 ± 3.1	6.1 ± 2.0	<0.001
Red Cell Distribution Width (RDW) (±SD) [%]	12.9 ± 0.8	12.4 ± 0.7	0.075
Mean Platelet Volume/Platelet Count Ratio (MPV/PC) (±SD)	0.045 ± 0.02	0.042 ± 0.01	0.465

SD: Standard Deviation.

## Data Availability

All data generated or analyzed during this study are included in this published article.

## Abbreviations

The following abbreviations are used in this manuscript:

CCM	Cerebral Cavernous Malformation
CrP	C-reactive Protein
WBC	White Blood Cell Count
RDW	Red Cell Distribution Width
MPV/PC	Mean Platelet Volume/Platelet Count Ratio
ROC	Receiver Operating Characteristic
MRI	Magnetic Resonance Imaging
AVM	Arteriovenous Malformation
SAH	Subarachnoid Hemorrhage
